# Adaptive Resistance to Immunotherapy Directed Against p53 Can be Overcome by Global Expression of Tumor-Antigens in Dendritic Cells

**DOI:** 10.3389/fonc.2014.00270

**Published:** 2014-10-06

**Authors:** Matjaz Humar, Marc Azemar, Martina Maurer, Bernd Groner

**Affiliations:** ^1^Pharmaceutical Biology and Biotechnology, Albert-Ludwigs-University of Freiburg, Freiburg, Germany; ^2^Internistische Onkologie, Tumor Biology Center, Freiburg, Germany; ^3^Basilea Pharmaceutica International Ltd., Basel, Switzerland; ^4^Institute for Biomedical Research, Georg Speyer Haus, Frankfurt am Main, Germany

**Keywords:** immune surveillance of cancer, tumor-specific antigen expressing dendritic cells, adaptive immune escape, dendritic cell–tumor cell fusion, CD8^+^ T-cell mediated tumor suppression

## Abstract

Immunotherapy of cancer utilizes dendritic cells (DCs) for antigen presentation and the induction of tumor-specific immune responses. However, the therapeutic induction of anti-tumor immunity is limited by tumor escape mechanisms. In this study, immortalized dendritic D2SC/1 cells were transduced with a mutated version of the p53 tumor suppressor gene, p53M234I, or p53C132F/E168G, which are overexpressed in MethA fibrosarcoma tumor cells. In addition, D2SC/1 cells were fused with MethA tumor cells to generate a vaccine that potentially expresses a large repertoire of tumor-antigens. Cellular vaccines were transplanted onto Balb/c mice and MethA tumor growth and anti-tumor immune responses were examined in vaccinated animals. D2SC/1–p53M234I and D2SC/1–p53C132F/E168G cells induced strong therapeutic and protective MethA tumor immunity upon transplantation in Balb/c mice. However, in a fraction of immunized mice MethA tumor growth resumed after an extended latency period. Analysis of these tumors indicated loss of p53 expression. Mice, pre-treated with fusion hybrids generated from D2SC/1 and MethA tumor cells, suppressed MethA tumor growth and averted adaptive immune escape. Polyclonal B-cell responses directed against various MethA tumor proteins could be detected in the sera of D2SC/1–MethA inoculated mice. Athymic nude mice and Balb/c mice depleted of CD4^+^ or CD8^+^ T-cells were not protected against MethA tumor cell growth after immunization with D2SC/1–MethA hybrids. Our results highlight a potential drawback of cancer immunotherapy by demonstrating that the induction of a specific anti-tumor response favors the acquisition of tumor phenotypes promoting immune evasion. In contrast, the application of DC/tumor cell fusion hybrids prevents adaptive immune escape by a T-cell dependent mechanism and provides a simple strategy for personalized anti-cancer treatment without the need of selectively priming the host immune system.

## Introduction

Progress in understanding the molecular basis of cancer etiology and insights into immunological defense mechanisms have led to promising new treatment options in the past decade (1–5). In addition to conventional chemotherapeutic agents, targeted compounds and immunotherapy have been added to the clinicians’ armamentarium. The enhancement of the immune system has been validated as a promising therapeutic strategy to elicit tumor-specific responses, to induce durable tumor regression, and to improve survival intervals of patients (6). However, objective clinical responses in patients subjected to immunotherapy are still insufficient. Benefits have only been observed in a fraction of patients and tumor-specific immune responses often did not correlate with tumor rejection, stabilization of disease, or overall survival (7). Considerable efforts are necessary to further improve immunotherapy of cancer and to gain insight into the complex interplay of tumor cells with the immune system (8, 9).

Tumor cells are poor antigen presenting cells. Therefore, the induction of protective immunity depends upon efficient tumor antigen (TA) presentation by professional antigen presenting cells (10). Activated DCs display surface antigens via major histocompatibility complexes (MHC) class I and class II in combination with co-stimulatory molecules, e.g., B7-1 and B7-2, and are able to interact with naïve CD4^+^ and CD8^+^ T-cells to trigger T-cell proliferation and differentiation (11, 12). Differentiated cytolytic CD8^+^ T-lymphocytes (CTLs) are the most important effector cells for anti-tumor immune responses (13). DCs are also able to interact with B-cells and thus stimulate specific antibody production (13). DCs have successfully been used as cancer vaccines by presentation of TAs (14).

However, therapeutic DC application is restricted by the limited number of known proteins, specifically expressed in tumor tissue and able to elicit an appropriate MHC-dependent immune response. For this reason, DCs have been pulsed with whole tumor lysates or have been fused with tumor cells to express and present multiple and unidentified TAs and to increase the frequency of responding immune effector cells (15, 16). The presentation of TAs in the context of abundant co-stimulation probably also avoids the induction of tolerance and promotes the polyclonal activation of helper T-cells and CTL responses (17). Furthermore, DCs are composed of multiple subsets, some of them essential for establishing tolerance through T-cell deletion and activation of regulatory T-cells (11). In contrast, CD8α^+^ DCs and DCs differentiated in medium with granulocyte macrophage colony-stimulating factor (GM-CSF) and IL-15 seem most suitable to induce antigen-specific CTLs (18, 19). The deployment of established DCs for analysis or comparison of antigen induced tumor responses could possibly circumvent potential problems, stemming from the logistics of primary cell culture, cytokine treatment, and the distinguishable properties of DC subsets. A cell line, D2SC/1 cells, has been established from mouse spleen, which exhibits dendritic cell (DC) properties and which was shown to be fully competent in bacterial or viral antigen presentation and the induction of primary T-cell responses (20–22).

The established fibrosarcoma tumor cell line MethA overexpresses two mutated versions of the p53 tumor suppressor gene, p53M234I, and p53C132F/E168G. Mutant p53 (Mp53) might contribute to the genesis of neo-antigens that provide epitopes exclusively expressed on the surface of tumor cells. Such T-cell determinants seem most suited for immune therapeutical interventions (23). In addition, p53 is expressed at nearly undetectable levels in normal tissues; however, mutations in p53 usually result in a prolonged half life and marked accumulation of mp53 has been observed in approximately 50% of human tumors. Mutated or overexpressed p53 are immunogenic. p53-derived epitopes in MHC-class I and MHC-class II complexes, MHC-class I and class II restricted p53-specific CTLs or CD4^+^ helper T-cells, and p53-specific circulating antibodies have been demonstrated (24–26). Experimental mouse models have used overexpression of p53 to induce immunogenicity and to achieve tumor rejection (27, 28). p53-specific immune responses have also been initiated by vaccination of cancer patients (29, 30).

The interaction of tumor cells with the immune system causes reciprocal evolutionary pressure. Tumors facilitate their own survival by formation of a cytokine microenvironment promoting angiogenesis and cell cycle progression and antagonize immune functions through pro-apoptotic signaling, prevention of efficient antigen processing and presentation, or the induction of immunosuppressive regulatory T-cells (14, 31). Another escape mechanism of cancer cells is based on adaptive resistance. Targeted treatment may lead to the reprograming of signaling networks and changes in gene expression patterns that result in resistance to particular drugs or loss of sensitivity against immune cells (32–34). This escape mechanism may potentially be blocked by the use of vaccines, which target all or most available antigens of a particular tumor cell. Vaccines which can compensate for the loss of a single TA may have a greater therapeutic potential (35).

We have compared the effectiveness of a DC vaccine based on the expression of a single TA, mp53, in D2SC/1 cells and a vaccine based on the presentation of a large number of potential antigens achieved through the fusion of MethA tumor cells with D2SC/1 DCs. Our experiments show that D2SC/1 cells can become potent inducers of anti-tumor immunity upon transfer of a mutated version of the p53 gene or the fusion with entire MethA tumor cells. An immune escape, seen in a fraction of D2SC/1–mp53 vaccinated mice, does not occur in mice vaccinated with the D2SC/1–MethA fusion cells. Fusion hybrids were more potent in stimulating anti-tumor immune responses because one single application was sufficient to induce immediate, comprehensive, and robust *in vivo* immune protection. Furthermore, TA-loaded D2SC/1 cells represent an attractive option to evaluate the immune stimulatory potential of diverse TAs.

## Materials and Methods

### Mice and cell lines

Female Balb/c (H-2d) mice were used at 6–8 weeks of age and purchased from Charles River (Sulzfeld, Germany). Female C57BL/6 (H-2b) and Balb/c athymic nude mice (H-2d) were obtained from Harlan Winkelmann (Borchen, Germany). All animal experiments were approved by the Regional Council of Freiburg and carried out in accordance with official regulations for care and use of laboratory animals.

MethA (H-2d) is a 3-methylcholanthrene induced fibrosarcoma, which arose in a Balb/c mouse (36). MethA tumor cells were cultured in RPMI 1640 medium, supplemented with 10% fetal calf serum. The HT1080-based packaging cell line FLY-AF-13 and the LacZ producer clone FLYA4lacZ3 were obtained from B. Schnierle, Langen, Germany (37). D2SC/1 (H-2d) are immortalized dendritic mouse cells isolated from spleen and were provided by U. Altenschmidt, Freiburg, Germany (21). FLY-AF-13, FLYA4lacZ3, and D2SC/1 cells were grown in Dulbecco’s modified Eagle medium and 10% fetal calf serum.

### Gene transfer by retroviral transduction

The open reading frames of the two mp53 alleles present in MethA tumor cells were cloned by RT-PCR, using the primer pair TCCGAAGCTTGGATGACTGC and GCAGAGGAATTCAGTCTGAGTCA. The missense point mutations C132F, E168G, and M234I present in the p53 alleles were verified by sequence analysis. p53M234I and p53C132F/E168G were cloned into the retroviral transduction vector pBABEpuro (Addgene, Cambridge, MA, USA). Stable amphotropic packaging cell lines were generated by calcium phosphate transfection of the mp53 vector constructs into the HT1080-based packaging cell line FLY-AF-13 and puromycin selection (5 μg/ml puromycin; Life Technologies, Darmstadt, Germany). Virus was obtained from producer cell lines at 40–60% confluence by replacing growth medium with 100 μl/cm^2^ RPMI 1640 medium, 10% FCS, and harvesting the conditioned medium 15 h later. Retroviral transduction was performed by filtering producer cell culture medium through a Pro-X™ 0.22 μM syringe filter (Roth, Karlsruhe, Germany) and adding it undiluted to 40% confluent, logarithmically growing D2SC/1 cells. Transduction was repeated at intervals of 15 h.

### Staining of LacZ transduced cells

Cells were fixed in 0.05% glutaraldehyde in phosphate buffered saline for 5 min at room temperature and stained in 137 mM NaCl, 2.7 mM KCl, 4.3 mM Na_2_HPO_4_, 1.4 mM KH_2_PO_4_, 2 mM MgCl_2_, 16 mM K_3_Fe(CN)_6_, and 16 mM K_4_Fe(CN)_6_ containing 1 mg/ml X-gal substrate (Sigma-Aldrich, St. Louis, MO, USA) for 6–48 h at 37°C. LacZ positive cells appeared blue under the microscope.

### Generation of cell fusion hybrids

The vector pBABEhygro (Addgene) was introduced into MethA tumor cells by calcium phosphate precipitation to obtain hygromycin B resistant clones. D2SC/1 cells were similarly transfected with pBABEpuro. Transfected cells were cultured in growth medium containing 5 μg/ml puromycin or 100 μg/ml hygromycin (Life Technologies). To obtain fusion hybrid cells, 10^7^ hygromycin resistant MethA tumor cells were mixed with 5 × 10^7^ puromycin resistant D2SC/1 cells and briefly centrifuged. Cellular pellets were gently resuspended in 1 ml PEG 4000 (Merck, Darmstadt, Germany) containing 0.5 ml RPMI 1640 medium and incubated at 37°C for 90 s. Subsequently, 15 ml RPMI 1640 medium was added drop wise to the cells, then 20 ml RPMI 1640 medium with 10% fetal calf serum. After 5 min the cell suspension was centrifuged and cells were plated in RPMI 1640 medium and 10% fetal calf serum. After 24 h the fused cell hybrids were selected in the presence of 100 μg/ml hygromycin and 5 μg/ml puromycin. When colonies were developed, individual hybrid cell clones were isolated using cloning cylinders (Sigma-Aldrich) and 50 μl of trypsin-EDTA.

### Immunization of mice

pBABE-LacZ, pBABE-p53M234I, or pBABE-p53C132F/E168G transduced D2SC/1 cells were irradiated with 50 gray and injected either intravenously (i.v.) into the tail vein or subcutaneously (s.c.) in the right flank of female Balb/c mice. 5 × 10^6^ mp53-transduced D2SC/1 cells were used for each injection. Injections were repeated five times in intervals of 10 days. Sixty days after onset of immunization mice were inoculated with 5 × 10^6^ non-irradiated MethA tumor cells by s.c. injection into the left flank.

Alternatively, 24 different cell hybrid clones, obtained by fusion of MethA tumor cells and D2SC/1 DCs were randomly chosen for immunization of female Balb/c mice or athymic Balb/c nude mice. 5 × 10^6^ non-irradiated hybrid cells were injected s.c. into the right flank of each mouse. After 4 weeks the immunized mice were inoculated with 5 × 10^6^ parental MethA tumor cells in the opposite flank. Tumor growth was monitored by palpation and calculated with vernier calipers based on the formula: tumor volume = (shortest diameter)^2^ × longest diameter/2. Tumor bearing mice were sacrificed on day 30 (s.c. tumor cell transplantation) or when animals displayed significant tumor necrosis (s.c. tumor cell transplantation), dyspnea, ataxia, weight loss, or lethargy (i.v. tumor cell application).

### Flow cytometry

Cell surface proteins were analyzed by a FACScan flow cytometer and the Lysis II software (BD Biosciences, Heidelberg, Germany). Briefly, 10^6^ cells were incubated with the specific primary antibody detecting B7-1 (clone 16-10A1), B7-2 (clone GL-1), H-2K[d] (SF1-1.1), H-2D[d] (clone 34-2-12), or I-A[d] (AMS-32.1) in 20 μl of phosphate buffered saline. After 45 min cells were washed and incubated with a fluorescein isothiocyanate (FITC) conjugated secondary antibody for 30 min. Antibody concentrations were used as indicated by the manufacturer (BD Biosciences). Free antibodies were removed by washing and samples were analyzed by flow cytometry in 500 μl of phosphate buffered saline.

For detection of intracellular p53, cells were suspended in 1 ml RPMI 1640 medium containing 50% fetal calf serum and fixed for 30 min on ice by drop wise addition of 3 ml 100% ethanol at −20°C. Then cells were washed with 3 × 10 ml phosphate buffered saline before an anti-p53 antibody (PAb 421, Millipore, Schwalbach, Germany) and a FITC conjugated secondary antibody (BD Biosciences) were added according to the recommendations of the manufacturer. Flow cytometry was performed in a volume of 500 μl phosphate buffered saline, after removing unbound antibodies by washing.

### Immunoblotting

MethA tumor cells were lysed in 150 mM NaCl, 10 mM Tris-HCl/pH 7.5, and 0.5% Triton X-100. Cell debris was removed by centrifugation at 13,000 × *g* for 5 min at 4°C, and protein concentrations of the cleared cellular lysates were determined by the bicinchoninic acid assay (Pierce, Rockford, IL, USA). The samples were mixed 1:1 with sample buffer (100 mM Tris-HCl/pH 6.8, 20% glycerol (v/v), 4% SDS, 200 mM dithiothreitol, and 0.02% bromphenol blue), boiled for 5 min, and 75 μg total protein of the cell lysate per centimeter was separated on a preparative 10% SDS-PAGE gel. Proteins were transferred to an Immobilon-P membrane (Millipore) that was cut to vertical stripes before non-specific binding sites were blocked for 1 h in blocking buffer (50 mM Tris-HCl/pH 7.6, 150 mM NaCl, 0.05% Tween 20, and 2.5% non-fat dry skim milk). Individual stripes were incubated for 1 h with mouse sera (1:400 in blocking buffer) and subsequently with a secondary horseradish-peroxidase (HRP)-labeled sheep anti-mouse IgG (1:5000; GE Healthcare, Little Chalfont, UK) for 40 min. MethA antibody complexes were visualized by the Amersham ECL™ Advance Western Blotting Detection Kit (GE Healthcare).

To detect endogenous p53, 75 μg of MethA tumor cell lysate were resolved by 10% SDS-PAGE, transferred to an Immobilon-P membrane, blotted with 1 μg/ml anti-p53 PAb 421 (Millipore) or β-actin antibody (1:1000, Cell Signaling Technology, Danvers, MA, USA) and specific bands were visualized by HRP-conjugated anti-mouse or anti-rabbit IgG and enhanced chemiluminescence reagents (GE Healthcare).

To detect p53-specific antibodies in mouse sera, the full open reading frame of mp53C132F/E168G or mp53M234I was cloned into the bacterial expression vector pRSET-C (Life Technologies) and after transformation of *E. coli* BL21-(DE3)LysS recombinant mp53 was purified by nickel-nitrilotriacetate column chromatography as previously described (38). Purified protein (100 ng/cm) was separated on preparative 10% SDS-PAGE gels and immobilized on Immobilon-P stripes that were individually blotted with mouse sera at a dilution of 1:400 or 1 μg/ml anti-p53 PAb 421 antibody. Mp53 antibody complexes were visualized by HRP-conjugated anti-mouse IgG and enhanced chemiluminescence reagents.

### Isolation of mouse T-cells and dendritic cells

T-lymphocytes from spleens of mice were isolated as described (39). Mice were sacrificed and the spleen was gently dissociated through a 200 μm mesh screen into a single cell suspension. Red blood cells were removed by lysis in 150 mM NH_4_Cl/pH 7.2, 1 M KHCO_3_, and 100 mM EDTA for 5 min. To enrich T-lymphocytes, spleen cell suspensions were passed through scrubbed nylon fiber wool columns that were pre-equilibrated with phosphate buffered saline and 10% fetal calf serum. Cells were allowed to adhere by incubating the loaded columns for 45 min at 37°C in a humidified incubator. Non-adherent cells were eluted in growth medium (Dulbecco’s modified Eagles medium, 10% fetal calf serum, 50 μM β-mercaptoethanol, 1 mM Hepes/pH 7.4, 100 μg/ml streptomycin, and 100 U/ml penicillin) and a fraction was analyzed by flow cytometry using an anti-CD3 phycoerythrin conjugated antibody according to the manufacturers description (clone 17A2, BD Biosciences). Eluted, non-attached cells were routinely 80–90% CD3^+^ T-cells.

Dendritic cells from spleen were enriched by positive selection using magnetic beads coupled to anti-mouse CD11c antibodies (clone N418, Miltenyi Biotec, Bergisch Gladbach, Germany). Briefly, 10^8^ spleen cells were suspended in 400 μl phosphate buffered saline, 10% fetal calf serum, and 2 mM EDTA and were co-incubated with 100 μl CD11c microbeads for 15 min at 4°C. Magnetically labeled cells were passed through a pre-equilibrated LS^+^ positive selection column, placed within a magnetic field of a MACS separator (Miltenyi Biotec). Unlabeled cells were removed by washing the column with 9 ml phosphate buffered saline, supplemented with 10% fetal calf serum and 2 mM EDTA. The column was removed from the magnetic field and CD11c positive cells were eluted as a positive selected cell fraction. DCs were maintained in growth medium containing Dulbecco’s modified Eagel’s medium, 10% fetal calf serum, 50 μM β-mercaptoethanol, 1 mM Hepes/pH 7.4, 100 μg/ml streptomycin, 100 U/ml penicillin, 5 ng/ml recombinant mouse IL-4 (Genzyme, Rüsselsheim, Germany), and 20 ng/ml recombinant mouse GM-CSF (Genzyme). Washed, adherent cells consisted of 90–98% DCs. Alternatively, DCs were obtained from bone-marrow by flushing the femurs of Balb/c mice as described previously (40). Bone-marrow derived cell suspensions were pre-cultured in DC growth medium for 10 days before differentiated cells were enriched by using CD11c microbeads and a MACS separator (Miltenyi Biotec).

### T-cell proliferation assay

T-cells were plated at 1.28 × 10^6^ cells per well of a 96 well microtiter plate or titrated 1:4 in triplicates. Proliferation of T-lymphocyte effector cells was induced by 2 × 10^4^ irradiated D2SC/1 cells, bone-marrow derived CD11c^+^ DCs, spleen derived CD11c^+^ DCs, or MethA-D2SC/1 fusion hybrid clones. Cells were co-cultured in 200 μl Dulbecco’s modified Eagle’s medium, 10% fetal calf serum, 100 U/ml penicillin, 100 μg/ml streptomycin, 10 mM Hepes/pH 7.4, 50 μM β-mercaptoethanol, and 0 4 ng/ml IL-2 (R&D Systems, Wiesbaden, Germany) for 72 h. One microcurie of [^3^H]thymidine (GE Healthcare) was added for the last 15 h. The labeled cells were harvested onto cellulose filters (Dunn Labortechnik GmbH, Asbach, Germany) and quenched (Ready Safe™, Liquid Scintillation Cocktail; Beckman Coulter, Krefeld, Germany). The amount of [^3^H]thymidine incorporation was determined by a liquid scintillation β-counter (LS 6500 Multi-Purpose Scintillation Counter; Beckman Coulter).

### *In vivo* depletion of CD4^+^ or CD8^+^ T-cells

Every second day, mice were treated by intraperitoneal injection with 100 μg of mAb GK1.5 (anti-CD4) or mAb 2.43 (anti-CD8) (Imgenex, San Diego, CA, USA) starting at 8 days before immunization of Balb/c mice with 5 × 10^6^ MethA–D2SC/1 fusion hybrid clones and challenge with 5 × 10^6^ parental MethA tumor cells. Depletion of the respective population by 40–80% was confirmed by flow cytometry using blood samples taken from the lateral tail vein and staining of peripheral blood leukocytes with anti-CD4 (GK1.5) FITC or anti-CD8 (2.43) PE (Santa Cruz Biotechnology, Santa Cruz, CA, USA) before MethA tumor challenge. Mice with ≤60% depletion of CD4^+^ or CD8^+^ T-cells were excluded from the experiment. MethA tumor growth was monitored by palpation and the size of the tumor was determined by vernier microcalipers.

### Statistical analysis

Statistical differences between experimental groups were determined by performing one-way analysis of variance followed by the Bonferroni’s *post hoc* test. Values are shown as ±standard error of the means. Differences between groups were considered to be significant at *p* < 0.05. Statistical analyzes were carried out using the Prism software package (GraphPad Software Inc., La Jolla, CA, USA).

## Results

### Generation of D2SC/1 dendritic cells expressing the mutated p53 variants found in MethA fibrosarcoma cells

**Figure 1:**
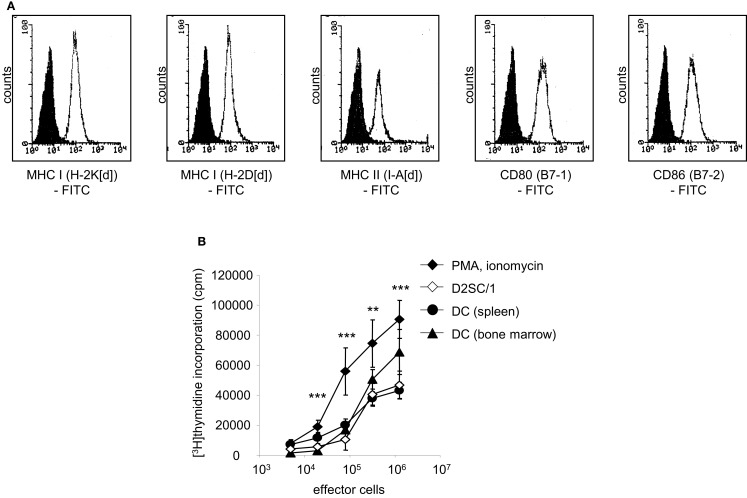
**D2SC/1 (H-2d) immortalized dendritic mouse splenocytes are potent stimulators of naïve T-cells**. **(A)** Flow cytometric analysis of D2SC/1 cells stained with anti-H-2K[d] (clone SF1-1.1), anti-H-2D[d] (clone 34-2-12), anti-I-A[d] (clone AMS-32.1), anti-B7-1 (clone 16-10A1), and anti-B7-2 (clone GL-1) antibodies. Filled histograms represent control staining without primary antibody (isotype control), open histograms the indicated surface marker. **(B)** A [^3^H]thymidine proliferation assay to determine T-cell activation. Allogeneic naïve T-cells from C57BL/6 mice were used as effector cells and stimulated with 50 ng/ml phorbol myristate acetate and 1 nM ionomycin (♦) or were co-cultured with 2 × 10^4^ irradiated D2SC/1 cells (◊), primary DCs derived from spleen (●), or primary DCs from bone-marrow (▲). Effector cells were stimulated for 72 h at 37°C and pulsed for the last 15 h with 1 μCi of [^3^H]thymidine. Values represent triplicate samples after subtracting thymidine incorporation in the absence of phorbol myristate acetate and ionomycin or stimulator cells. Data are shown as ±standard error of the means of three independent experiments using CD3^+^ T-lymphocytes and primary DCs of different donors. Experimental groups were statistically evaluated by performing one-way ANOVA followed by the Bonferroni’s *post hoc* test. Statistical differences of alloresponsive D2SC/1 cells was only observed versus phorbol myristate acetate and ionomycin treated cells (**p < 0.01; ***p < 0.001).

We analyzed the potential of immortalized dendritic D2SC/1 cells to induce an effective cellular immune response. D2SC/1 cells express the antigen presenting molecules MHC-class I and MHC-class II and the co-stimulatory molecules B7-1 and B7-2 (Figure 1A) and are able to induce a strong allogeneic immune response (Figure 1B). 2 × 10^4^ DCs/well caused a marked stimulation of the proliferation of allogeneic naïve T-lymphocytes isolated from C57/BL6 mice. D2SC/1 cells were nearly as efficient in stimulating T-cell proliferation as primary DCs isolated from mouse spleen or differentiated from bone-marrow cells (Figure 1B). The capacity of D2SC/1 cells to stimulate T-cell activation is a necessary prerequisite for its function as a cellular vaccine and its application in experimental tumor therapy.

**Figure 2:**
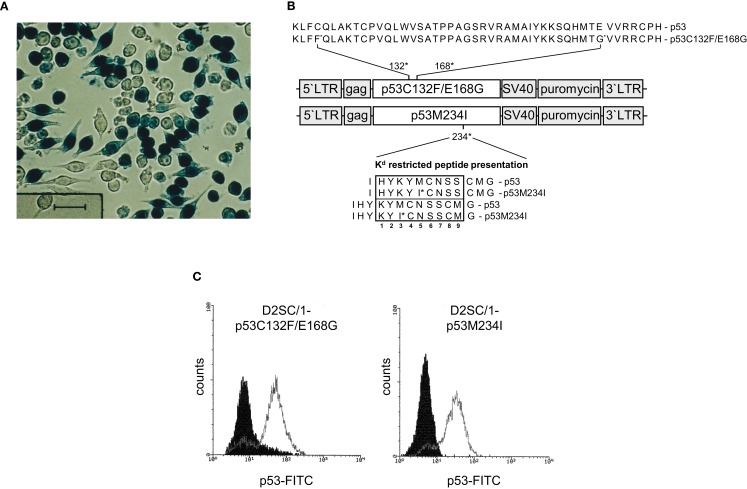
**Retroviral transduction of D2SC/1 cells**. **(A)** D2SC/1 cells were cultured in the supernatant of the retroviral packaging cell line FLYA4lacZ3 for 24 h and LacZ transduced cells were visualized by X-gal staining. **(B)** The mp53 retroviral transduction vectors are illustrated. Predicted K[d] restricted peptide presentation is marked by a box. Amino acids at position 2, 3, 5, and carboxyl termini function as anchors or auxiliary anchors within the MHC pocket. **(C)** Flow cytometry of mp53-transduced D2SC/1. Stable amphotropic packaging cell lines were generated by calcium phosphate transfection of FLY-AF-13 cells with pBABEpuro–p53C132F/E168G or pBABEpuro–p53M234I and puromycin selection before D2SC/1 cells were transduced three times with 1.5–3 × 10^4^ CFU/ml of budding virus for 15 h. Cells were fixed and stained with 1 μg/ml PAb 421 anti-p53 antibody. Filled histograms represent stained cells transduced with the pBABEpuro control vector, open histograms D2SC/1-p53C132F/E168G, or D2SC/1-p53M234I transduced cells.

Gene transfer of TAs into DCs predetermines the presentation of the transgene via the MHC-class I pathway and induction of cytolytic antigen-specific target cells (41). Unfortunately, standard transfection methods such as calcium phosphate precipitation, lipofection, and electroporation did not allow the efficient transfer of transgenes into D2SC/1 cells and primary DCs isolated from mouse spleen or differentiated from bone-marrow (data on file). For this reason, we established the conditions for retroviral gene transfer and initially introduced the LacZ gene. D2SC/1 cells were cultured in medium obtained as supernatants from the packaging cell line FLYA4lacZ3 and thus infected with LacZ encoding viruses (37). Efficient gene transfer was observed and more than 70% of the D2SC/1 cells displayed expression of the LacZ transgene (Figure 2A).

We supplied D2SC/1 cells with mutant versions of the p53 gene present in MethA cells to construct an efficient cellular anti-tumor vaccine. Two mutant alleles of p53 (p53M234I and p53C132F/E168G) were cloned from the methylcholanthrene induced Balb/c sarcoma cell line MethA by RT-PCR and introduced into the retroviral transduction vector pBABEpuro (Figure 2B). The presence of the missense point mutations C132F, E168G, or M234I in the vectors were confirmed by sequence analysis (data on file). Stable amphotropic packaging cell lines were generated by calcium phosphate transfection of p53M234I or p53C132F/E168G vector constructs into the HT1080-based packaging cell line FLY-AF-13 and subsequent puromycin selection. Conditioned medium from the stably transfected packaging cell lines, containing the mp53 encoding retroviruses, was added to logarithmically growing D2SC/1 cells. Multiple infections were performed to increase the transduction efficiency. After three cycles of infection, transduction efficiency was found to be between 75 and 98% as determined by flow cytometry (Figure 2C). Two days post transduction cells were harvested, irradiated, and used for the vaccination of Balb/c mice.

### Mutant p53 expressing D2SC/1 cells, supplied as a cellular vaccine, induces immunity to the growth of transplanted MethA tumor cells

**Figure 3:**
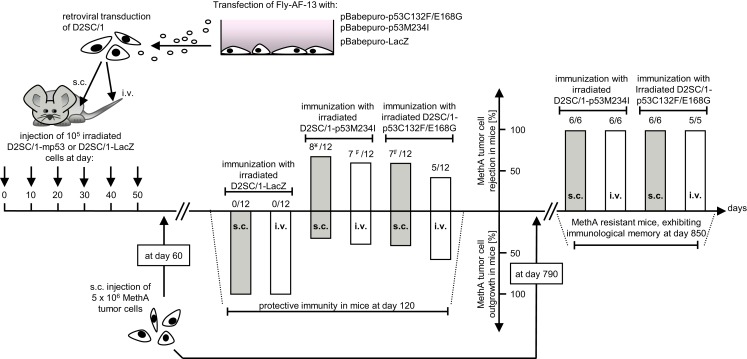
**Immunization protocol of Balb/c mice treated with transduced D2SC/1 cells**. Stable amphotropic packaging cell lines were generated by calcium phosphate transfection of FLY-AF-13 cells with pBABEpuro–p53C132F/E168G or pBABEpuro–p53M234I and puromycin selection before D2SC/1 cells were transduced with 1.5–3 × 10^4^ CFU/ml of budding virus for three times and irradiated with 50 grays. Mice received 5 × 10^6^ transduced and irradiated D2SC/1 cells s.c. in the right flank or i.v. in the tail vain every 10 days. Sixty days after onset of immunization, recipient mice were inoculated with 5 × 10^6^ MethA tumor cells by subcutaneous injection into the left flank. MethA tumor resistance of mice was accessed at day 120. The amount of tumor free mice and the administered cellular vaccine are indicated within the diagram. Tumor resistant mice were re-challenged with 5 × 10^6^ MethA tumor cells in the left flank after an additional 2 years. Delayed tumor growth 3 months after tumor cell transplantation is marked by ¥ (in two mice) or ₣ (in one mouse).

D2SC/1 cells are immortalized DCs. To prevent their outgrowth after injection into mice, they were irradiated with 50 gray before their use as a cellular vaccine. The mice inoculated with the irradiated cells were monitored for up to 15 months and no growth of the D2SC/1 cells was observed. The vaccination protocol is shown in Figure 3. 5 × 10^6^ irradiated D2SC/1 cells were injected either intravenously into the tail vein or subcutaneously into the right flank of the animals. To boost the immune response, injections were repeated five times in intervals of 10 days. Sixty days after the first injection with D2SC/1 cells, mice were inoculated with 5 × 10^6^ MethA tumor cells by subcutaneous injection into the opposite, left flank. Tumor growth was monitored by palpation and the size of the tumors was determined by vernier microcalipers. The data obtained 2 months after tumor cell implantation are shown in Figure 3 and Table 1. Mice treated with irradiated D2SC/1 cells that were infected with the control vector pBABEpuro harboring the LacZ gene, developed no protective immunity and displayed unrestricted MethA tumor growth. In contrast, mice inoculated with irradiated D2SC/1 cells that were infected with the viral vector encoding mp53, were protected from MethA tumor growth in 27 out of 48 cases. Inhibition of tumor growth was observed when both mp53 alleles were expressed in the D2SC/1 cells, independent of the application route of the cellular vaccine. In the non-protected, D2SC/1-mp53 vaccinated mice, the growth kinetics of MethA tumors were identical to those observed in D2SC/1-LacZ inoculated control mice (Figure S1 in Supplementary Material).

**Table 1:** **Number of mice protected from MethA tumor growth after immunization with irradiated mp53-transduced D2SC/1 cells**.

Cellular vaccine	Subcutaneous	Intravenous
	injection of	injection of
	transduced D2SC/1	transduced D2SC/1
D2SC/1-p53C132F/E168G	7^b^ (*n* = 12)	5 (*n* = 12)
D2SC/1-p53M234I	8^a^ (*n* = 12)	7^b^ (*n* = 12)
D2SC/1-LacZ	0 (*n* = 12)	0 (*n* = 12)

*^a^Delayed MethA tumor growth 3 months post MethA tumor cell inoculation in two mice*.

*^b^Delayed MethA tumor growth 3 months post MethA tumor cell inoculation in one mouse*.

*Immunization was performed as described in Figure 3. Data obtained 3 months after first MethA tumor cell transplantation are summarized*.

Two years later, mice were exposed a second time to MethA tumor cells. All of the 23 vaccinated mice which had not developed tumors after the first round of tumor cell inoculation rejected the transplanted tumor cells also in the second round. This indicates the establishment of a long term immunological memory (Figure 3).

**Figure 4:**
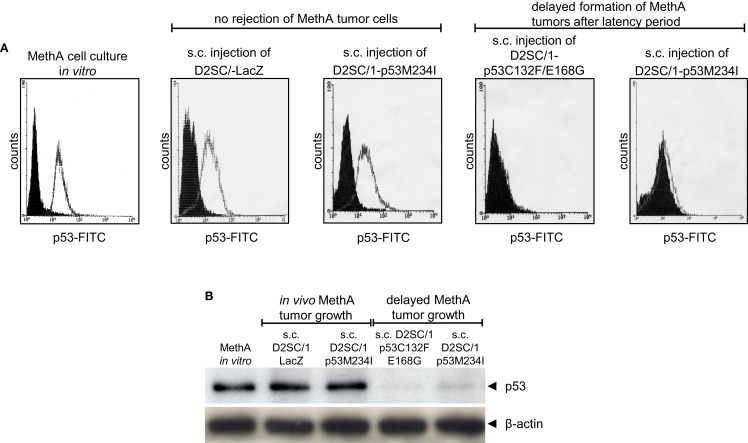
**Outgrowth of p53 negative MethA tumor cells in mice that were immunized by irradiated D2SC/1–mp53**. MethA tumors were excised from tumor bearing mice and single cell suspensions were assayed for p53 expression by flow cytometry **(A)** or immunoblotting **(B)**. Parental, *in vitro* cultured MethA cells and MethA tumor cells isolated from mice that were treated with irradiated LacZ transduced D2SC/1 cells served as controls. The immunization procedure and tumor appearance are indicated at the top of each image. The β-actin antibody was used as a loading control.

After the first tumor cell inoculation, four D2SC/1–mp53 vaccinated mice that initially displayed no MethA tumor growth, developed tumors with a delay of 2–3 months at the MethA cell transplantation site. We hypothesized that the vaccination of the mice with D2SC/1–mp53 cells caused a selective pressure on the tumor and that tumor cells that grew out after a latency period might have acquired a p53 negative phenotype preventing immunological recognition. The late appearing tumors were excised, explants were mechanically dissociated by a 200 μm mesh screen, and the resultant single cell suspensions were examined by flow cytometry or immunoblotting to test the possibility that a subset of tumor cells had downregulated their mp53 expression (Figure 4). MethA tumor cells isolated from mice vaccinated with D2SC/1–LacZ cells or the parental *in vitro* cultured MethA cell line served as controls and displayed high expression of p53. P53 levels from controls were comparable to those found in samples from tumor biopsies isolated from D2SC/1–mp53 vaccinated mice, when tumor growth was not affected by the cellular vaccine. However, no p53 expression was found in tumor cells isolated from D2SC/1–mp53 vaccinated mice that were derived from tumors with delayed appearance. This observation was independent of the mp53 allele used in the vaccination protocol. Delayed tumor growth in DC immunized mice is most likely the result of adaptive resistance, the downregulation of p53 expression resulting in the outgrowth of escape variants.

### Fusion of D2SC/1 cells with MethA tumor cells

**Figure 5:**
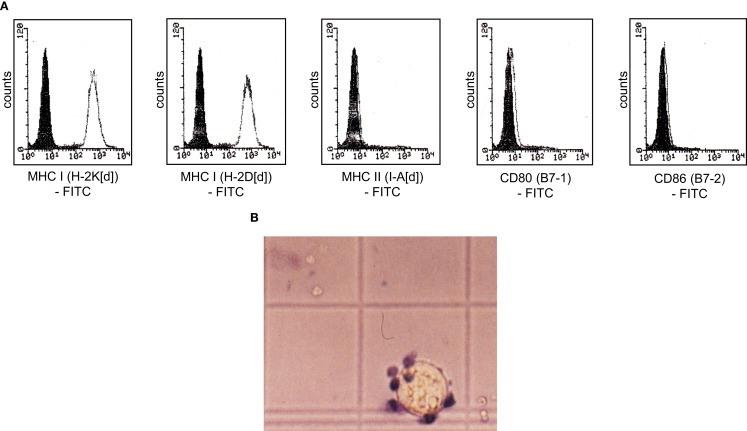
**MethA tumor cells are resistant to T-cell activation *in vitro***. **(A)** Flow cytometric analysis of MethA tumor cells stained with anti-H-2K[d] (clone SF1-1.1), anti-H-2D[d] (clone 34-2-12), anti-I-A[d] (clone AMS-32.1), anti-B7-1 (clone 16-10A1), and anti-B7-2 (clone GL-1) antibodies. Filled histograms represent control staining without the primary antibody (isotype control), open histograms the indicated surface marker. **(B)** T-cells isolated from C57/BL6 mice and MethA tumor cells were co-cultured in proliferation medium (DMEM, 10% FCS, 10 mM HEPES/pH7.4, 50 μM ß-mercaptoethanol, 0.4 ng/ml IL-2, 100 U/ml penicillin, and 100 μg/ml streptomycin) for 3 days and analyzed by a trypan blue exclusion assay. A vital MethA tumor cell is shown that excludes the dye, whereas, bound T-lymphocytes are intensively stained.

Native MethA tumor cells are most likely only weakly immunogenic, they express the MHC-class I alleles H-2K[d] and H-2D[d], but not the MHC-class II allele I-A[d] and the co-stimulatory molecules B7-1 and B7-2 (Figure 5A). MHC-class I associated TA presentation is necessary for the interaction with cytotoxic T-lymphocytes via the T-cell receptor and the induction of cellular immunity. However, lack of co-stimulatory signals renders T-cells tolerant and induces anergy or even cell death (42). We observed the binding of alloreactive T-lymphocytes to MethA tumor cells *in vitro*, but trypan blue exclusion assays revealed that the bound T-cells were not vital, whereas, MethA tumor cells excluded the dye (Figure 5B). Furthermore, naïve T-lymphocytes did not proliferate in the presence of IL-2 or phorbol myristate acetate and ionomycin when exposed to MethA cell culture supernatants (data on file).

D2SC/1 cells express high levels of B7-1, B7-2, MHC-class I, and MHC-class II molecules on their cell surface (Figure 1). These co-stimulatory molecules are responsible for the potency of these cells as professional APCs and their capacity to stimulate anti-tumor T-cells. Table 1 and Figure 3 show that TA expressing D2SC/1 cells confer protective immunity *in vivo*, but that the anti-tumor immune response can be impaired by the downregulation of mp53, the applied TA (Figure 4).

**Figure 6:**
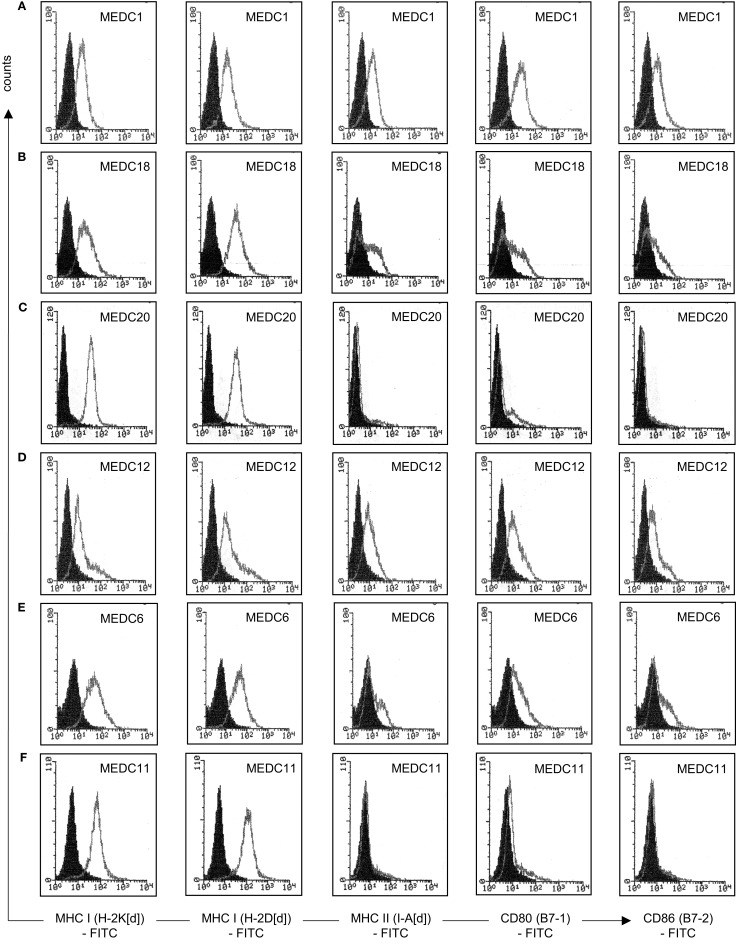
**Characterization of individual D2SC/1-MethA fusion hybrids by flow cytometry**. Expression of MHC complexes and B7 co-stimulatory molecules of clone MeDC1 **(A)**, MeDC18 **(B)**, MeDC20 **(C)**, MeDC12 **(D)**, MeDC6 **(E)**, and MeDC11 **(F)** were analyzed with anti-H-2K[d] (clone SF1-1.1), anti-H-2D[d] (clone 34-2-12), anti-I-A[d] (clone AMS-32.1), anti-B7-1 (clone 16-10A1), and anti-B7-2 (clone GL-1) antibodies. Filled histograms represent control staining without the primary antibody (isotype control), open histograms the indicated surface marker. Clones that mediate MethA tumor rejection are MeDC1, MeDC18, and MeDC20. Clones that remain tumorigenic are MeDC11 and MeDC12. Clone MeDC6 does not mediate protective anti-tumor immunity, although it has lost tumorigenicity.

The fusion of tumor cells with D2SC/1 cells can result in a cellular vaccine that is potentially able to present the majority of TAs characteristic for MethA cells and combine them with the DC capabilities of processing, presentation, and immune stimulation. The provision of vaccines presenting a wide range of tumor cell epitopes can potentially enhance selective pressure and prevent the evolution of tumor cell escape variants. For generation of the fusion hybrids, we stably transfected D2SC/1 with a puromycin and MethA tumor cells with a hygromycin B selection marker. Resistant clones of both cell lines were isolated and fused through exposure to polyethylene glycol. The fusion products were selected through the simultaneous exposure of the cells to puromycin and hygromycin B and 56 individual cell hybrid clones were isolated. Twenty-four hybrid clones were further characterized. Flow cytometric analyses indicated the expression of MHC complexes and the co-stimulatory molecules B7-1 and B7-2 (Table 2, Figure 6). Most of the analyzed clones expressed all of the surface proteins tested, although the density of MHC I, MHC II, B7-1, and B7-2 varied in individual hybrid clones. Characterized clones were subsequently used for the vaccination of mice and the animals were challenged with MethA tumor cells.

**Table 2:** **Characterization of D2SC/1–MethA fusion hybrid clones and their potential as a cellular vaccine**.

Fusion hybrid	Expression of H-2					Rejection of fusion hybrid in		Protective immunity in Balb/c mice against		Immunologic
ID number						Balb/c or Balb/c nude		solid tumor growth or metastasis of MethA		memory in Balb/c
	K[d]	D[d]	A[d]	B7-1	B7-2	
MeDC1	++	+++	++	+++	++	Yes	No	Yes	Yes	Yes
MeDC2	+++	+++	−	+	−	Yes	No	Yes	Yes	Yes
MeDC3	+++	+++	−	−	−	Yes	No	Yes	Yes	Yes
MeDC4	++	+++	−	+	−	Yes	No	Yes	Yes	Yes
MeDC5	++	++	++	++	+	Yes	No	Yes	Yes	Yes
MeDC6	++	++	+	+	+	Yes	No	No	No	n.d.
MeDC7	++	++	+	++	+	Yes	No	Yes	Yes	Yes
MeDC8	++	++	−	+	−	Yes	No	Yes	Yes	Yes
MeDC9	++	++	++	++	+	Yes	No	Yes	Yes	Yes
MeDC10	+++	+++	−	−	−	Yes	No	Yes	Yes	Yes
MeDC11	+++	+++	−	−	−	No	No	n.d.	n.d.	n.d.
MeDC12	++	++	++	++	+	No	No	n.d.	n.d.	n.d.
MeDC13	+++	+++	++	++	+	Yes	No	Yes	Yes	Yes
MeDC14	+	++	+	++	+	No	No	n.d.	n.d.	n.d.
MeDC15	++	++	−	−	−	Yes	No	Yes	Yes	Yes
MeDC16	++	++	+	+	+	No	No	n.d.	n.d.	n.d.
MeDC17	++	++	+	+	−	Yes	No	Yes	Yes	Yes
MeDC18	++	+++	+	+	−	Yes	No	Yes	Yes	Yes
MeDC19	+++	+++	++	++	++	Yes	No	Yes	Yes	Yes
MeDC20	+++	+++	−	−	−	Yes	No	Yes	Yes	Yes
MeDC21	++	+	+	++	+	No	No	n.d.	n.d.	n.d.
MeDC22	++	++	+	++	++	Yes	No	Yes	Yes	Yes
MeDC23	++	++	++	++	+	Yes	No	Yes	Yes	Yes
MeDC24	+++	+++	−	+	−	Yes	No	Yes	Yes	Yes

*(−) increase in expression by <25%, (+) by 25–50%, (++) by 50–80%, or (+++) by more than 80%; n.d., not determined*.

*Cell surface marker expression was analyzed by flow cytometry. Additionally, rejection of individual transplanted fusion hybrid clones or MethA tumor cells is shown. Immunologic memory was proven by a second rejection of MethA tumor cells, transplanted to mice 2 years after the first MethA tumor cell inoculation*.

### Vaccination of mice with D2SC/1-MethA cell hybrids

5 × 10^6^ D2SC/1-MethA cells derived from of a single hybrid clone were injected subcutaneously in the right flank of Balb/c mice. One week after the inoculation, small nodules of 2–3 mm in diameter formed at the site of injection as hybrid clones were not irradiated before their application and D2SC/1 or MethA tumor cells form aggressive tumors when injected into mice. These nodules progressively diminished within 2 weeks, indicating the induction of a specific immune response (data on file). However, 5 out of 24 D2SC/1–MethA hybrid clones developed tumors (Table 2). The aggressiveness of these five clones could not be correlated with the expression profile of MHC complexes or the B7-1 and B7-2 co-stimulatory molecules (Table 2; Figure 6).

Four weeks after the vaccination with D2SC/1–MethA cells, the remaining 19 mice were challenged with 5 × 10^6^ parental MethA tumor cells. The cells were injected subcutaneously in the opposite left flank to monitor potential tumor growth. Alternatively, 5 × 10^6^ MethA tumor cells were injected intravenously into the tail veins of the mice to determine the possible formation of lung metastasis (27). Table 2 summarizes the results of these experiments. Fusion of D2SC/1 cells with MethA tumor cells produced hybrid cells that have largely lost their ability to form tumors. These fusion hybrids assumed the role of a potent vaccine and quantitatively protected mice, after only a single application of the cells, from growth of MethA tumor cells. MethA tumor cells transiently formed small nodules at the site of injection in treated mice; however, these nodules regressed within 2 weeks when MethA cells were supplied to D2SC/1–MethA vaccinated animals. We conclude that a specific immune response deleted the MethA tumor cells.

Mice, immunized with individual hybrid variants were also protected from lung metastasis formed by MethA cells upon tail vein injection (Table 2). Control mice, not treated with the cellular vaccine, exhibited unrestricted MethA tumor growth and developed metastases in the lung (data on file). Vaccinated, tumor resistant mice were exposed to a second challenge with MethA cells 24 months later. All animals rejected the transplanted tumor cells. This indicates a long-lasting immunological memory (Table 2).

### Immune effector mechanisms

The induction of systemic anti-tumor immunity and the establishment of immunological memory are the characteristic features of adaptive immune responses. We observed that mice, transplanted with the cellular D2SC/1 vaccines at distinct sites, responded to the second challenge with MethA tumor cells 2 years after the initial immunization and exhibited resistance against tumor and metastasis formation (Table 2). Therefore, we analyzed the mice vaccinated with D2SC/1–mp53 cells or D2SC/1–MethA cells for the presence of major effectors of the adaptive immune response, characteristic for immunological memory.

**Figure 7:**
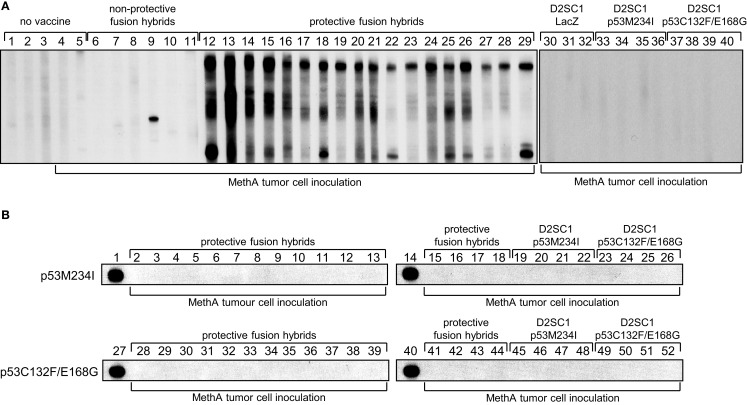
**Polyclonal B-cell activation by D2SC/1–MethA fusion hybrids**. Balb/c mice were vaccinated with 5 × 10^6^ transduced and irradiated D2SC/1 cells or non-irradiated fusion hybrid clones and inoculated s.c. with 5 × 10^6^ MethA tumor cells. Blood sera were isolated 10 days post tumor cell transplantation and analyzed by immunoblotting at a dilution of 1:400 using blotted MethA tumor cell lysates **(A)** or purified mp53 **(B)**. Donor mice are indicated by numbers and vaccine at the top of the blots, MethA tumor cell inoculation is displayed at the bottom of the blots. Pre-immune serum (A1–3), sera from mice 10 days post MethA tumor cell transplantation (A4–5), sera from fusion hybrid vaccinated mice that do not mediate MethA rejection: MeDC16 (A6), MeDC21 (A7), MeDC6 (A8), MeDC11 (A9), MeDC14 (A10), and MeDC12 (A11), sera from fusion hybrid vaccinated mice that mediate MethA rejection: MeDC7 (A12, B2, B28), MeDC24 (A13, B3, B29), MeDC22 (A14, B4, B30), MeDC13 (A15, B5, B31), MeDC4 (A16, B6, B32), MeDC2 (A17, B7, B33), MeDC8 (A18, B8, B34), MeDC3 (A19, B9, B35), MeDC9 (A20, B10, B36), MeDC15 (A21, B11, B37), MeDC10 (A22, B12, B38), MeDC23 (A23, B13, B39), MeDC5 (A24, B15, B41), MeDC17 (A25, B16, B42), MeDC1 (A26, B17, B43), MeDC19 (A27, B18, B44), MeDC20 (A28, B19, B45), and MeDC18 (A29, B20, B46), sera from D2SC/1–LacZ vaccinated mice after MethA transplantation (A30–32), sera from D2SC/1–p53M234I vaccinated mice after MethA transplantation (A33–36, B21–23, B47–49), and sera from D2SC/1–p53C132F/E168G vaccinated mice after MethA transplantation (A37–40, B24–26, B50–52), positive control using 1 μg/ml anti-p53 PAb 421 antibody (B1, B14, B27, B40).

Dendritic cells stimulate growth and differentiation of antigen-specific B-lymphocytes (43). To investigate whether D2SC/1 inoculated mice express TA specific antibodies, we obtained serum from Balb/c mice vaccinated with D2SC/1–LacZ, D2SC/1–mp53, and D2SC/1–MethA. Blood samples were taken from the lateral tail vein before and after vaccination. The sera were tested for the presence of MethA specific antibodies by exposure to membranes onto which MethA tumor cell lysates or purified mp53 were immobilized (Figure 7). Our results show that only antibodies present in sera from mice vaccinated with D2SC/1–MethA, which had rejected the MethA tumor cell challenge, recognized multiple MethA tumor cell proteins. These antibodies were not found in pre-immune sera or in sera from mice vaccinated with D2SC/1–mp53. Also, sera from control mice inoculated with MethA tumor cells without D2SC/1 vaccination, from mice vaccinated with D2SC/1–lacZ or from mice vaccinated with D2SC/1–MethA that were not protected against the MethA tumor cell challenge, did not contain antibodies of this specificity. These results show that MethA reactive antibodies are specifically induced in mice, upon vaccination with D2SC/1–MethA, which are able to suppress tumor cell growth and indicate that these antibodies might be involved in this process.

We evaluated the relative contribution of B-lymphocytes to the anti-tumor response in our mouse model. Athymic nude mice were vaccinated by subcutaneous injection of non-irradiated D2SC/1–MethA hybrid cells. The athymic nude mice are characterized by a defective T-cell response and immune responses in these animals are independent of mature T-lymphocytes. We observed aggressive tumor growth upon transfer of D2SC/1–MethA hybrid cells into the athymic nude mice. These tumors were not eliminated by the immune system (Table 2). We also observed that the growth kinetics of individual fusion clones varied substantially (data on file). This indicates that individual clones possess different potentials for tumorigenicity, possibly reflected in distinguishable gene expression patterns (Table 2; Figure 6). We also conclude that D2SC/1–MethA hybrid cells retain their tumorigenicity in the absence of T-lymphocytes. It is conceivable that co-stimulatory CD4^+^ T-cells may assist B-lymphocyte activation and the induction of humoral immunity.

### MethA tumor rejection is mediated by T-lymphocytes

**Figure 8:**
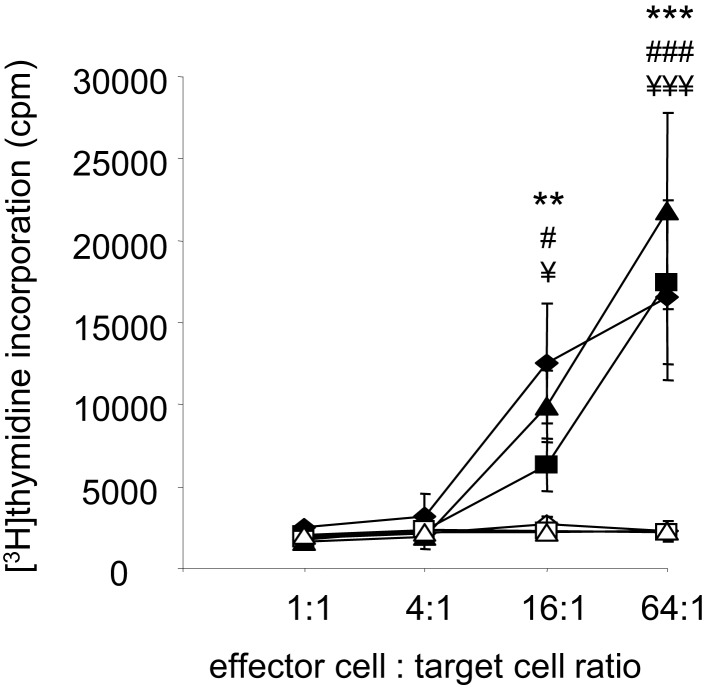
**Fusion hybrid dependent T-cell activation**. Fusion hybrid specific T-lymphocytes were detected by a [^3^H]thymidine incorporation assay. Balb/c mice were vaccinated with 5 × 10^6^ non-irradiated fusion hybrids, inoculated with 5 × 10^6^ MethA tumor cells and dissected to isolate spleen derived T-lymphocytes. 1.28 × 10^6^ T-cells were titrated by a fourfold dilution before proliferation was induced by co-culture with 2 × 10^4^ irradiated (200 gray) hybrid fusion clones for 72 h. One microcurie [^3^H]thymidine was added for the last 15 h of the experiment. Clones used for vaccination and *in vitro* target cells are MeDC1 (♦), MeDC18 (▲), and MeDC20 (■). In control experiments, effector T-cells from non-vaccinated, 5 × 10^6^ MethA tumor cell inoculated mice were similarly exposed to 2 × 10^4^ irradiated MeDC1 (◊), MeDC18 (Δ), or MeDC20 (□) cells *in vitro*. Values represent the ±standard error of the means (*n* = 3) after subtracting thymidine incorporation in the absence of stimulator cells. Statistical differences using MeDC1 (**p < 0.01; ***p < 0.001), MeDC18 (^###^*p* < 0.001), or MeDC20 (^¥^*p* < 0.05; ^¥¥¥^*p* < 0.001) target cells were determined by performing one-way ANOVA followed by the Bonferroni’s *post hoc* test.

Despite the potential involvement of B-cells and MethA specific antibodies, we hypothesize that differentiated cytotoxic CD8^+^ T-lymphocytes are the critical mediators of the induced anti-tumor immunity. They are most likely activated by MHC-class I TA peptide complexes on the surface of the fusion hybrids and are then able to lyse the tumor cells and the observed growth of the D2SC/1–MethA hybrid clones in athymic nude mice supports this notion. To corroborate this observation, we directly investigated whether vaccination with D2SC/1–MethA fusion hybrids induces a specific T-cell response. Balb/c mice resistant against the challenge with MethA tumor cells were used and T-lymphocytes from their spleens were isolated. T-cells from non-immunized mice, challenged with MethA tumor cells served as controls. The T-cells were co-cultured with D2SC/1–MethA cells and their proliferative response was measured. Our results show that T-cells from protected, vaccinated animals can be activated in the presence of fusion hybrids (see Figure 8). T-cells from unprotected control animals were not growth stimulated. D2SC/1 cells, used as target cells, were not able to elicit the proliferative effect, i.e., the T-cell recognized the MethA tumor cells (data on file).

**Figure 9:**
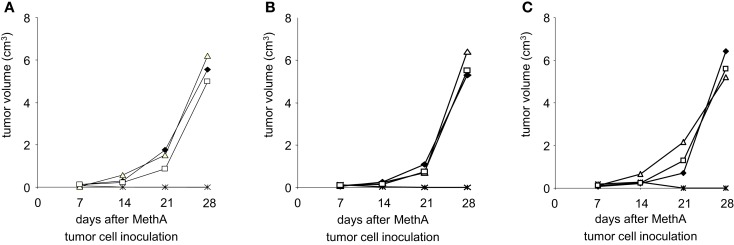
**Depletion of T-lymphocytes results in loss of fusion hybrid induced anti-tumor immunity**. MethA tumor growth is shown in CD4-depleted (Δ) or CD8-depleted (□) Balb/c mice. T-cell depleted mice were vaccinated with 5 × 10^6^ MeDC1 **(A)**, MeDC18 **(B)**, or MeDC20 **(C)** 28 days before inoculation with 5 × 10^6^ MethA tumor cells. ♦, MethA tumor growth in non-vaccinated, non-depleted CD4^+^/CD8^+^ Balb/c mice. *, MethA tumor growth in fusion hybrid vaccinated, non-depleted CD4^+^/CD8^+^ Balb/c mice.

To further define the effectors responsible for the anti-tumor cell immunity, CD4^+^ or CD8^+^ T-lymphocytes were depleted from Balb/c mice by injection of either anti-CD4 or anti-CD8 specific antibodies. Such animals were then vaccinated with D2SC/1–MethA fusion hybrids and challenged with MethA tumor cells. Our results show (Figures 9A–C) that the protection from MethA induced tumor growth requires both CD4^+^ and CD8^+^ T-cells. The depletion of CD4^+^ or CD8^+^ T-lymphocytes abrogated the protective effect of the vaccination. We conclude that a CD4^+^-assisted B-lymphocyte response and a robust activation of tumor-specific cytotoxic T-cells might cooperate in the anti-tumor cell immunity, induced by the D2SC/1–MethA cells.

## Discussion

Cellular immune therapy of cancer is well on its way into clinical practice. Vaccine-induced expansion of antigen-specific effector T-cells and clinical responses have been observed in the treatment of vulvar intraepithelial neoplasia and treatment of metastatic melanoma patients with ipilimumab improved their survival (44, 45). Clinical responses, however, were not always satisfactory. Benefits were only observed in a fraction of patients and TA specific immune responses often did not correlate with tumor rejection, stabilization of disease, or overall survival (7). The procedures of immune therapy still have to be optimized. Counteracting tumor evasion mechanisms, the identification and utilization of new TAs and the design of innovative vaccination protocols could pave the way.

Our study contributes two potentially valuable aspects: (1) immortalized D2SC/1 DCs can efficiently be used to present immunogenic epitopes of mutated p53 variants after retroviral transduction and induce a robust rejection of MethA fibrosarcoma cells. We also observed that the MethA tumor cells can evolve under the pressure exerted by immune cells and downregulate the expression of mutated p53 alleles. This results in the outgrowth of MethA escape variants. (2) A more comprehensive and sustained immune protection was achieved when D2SC/1 cells were fused with MethA and the hybrid cells were employed as vaccines. The hybrid cells combine the expression of a large variety of MethA specific genes and the processing and presentation capabilities of DCs. These cells are able to induce a protective immune response, which cannot easily be subverted by escape variants of the tumor cells.

The p53 tumor suppressor seems an attractive candidate as a functional TA (46, 47). p53 is found to be mutated in about 50% of all human tumors and the mutated protein accumulates in the cancer cells. p53 mediated signaling pathways are abnormal in nearly all human malignancies. Although the mutations in the p53 gene can occur at different positions, hotspots have been identified and immune responses to mutated or overexpressed p53 have been found. p53-specific epitopes can be presented by MHC-class I and MHC-class II molecules and p53-specific CD8^+^ CTLs or CD4^+^ T-helper cells have been detected as well as p53-specific circulating antibodies (24–26). p53 has shown its value as a target in immune therapy in animal models and in patients (27, 30).

The selection of suitable TAs in immune therapy has to be complemented by proper ways of administration to achieve sustained anti-tumor immunity. DCs seem the route of choice (14, 18). They are able to degrade and present immunogenic peptides in the context of MHC molecules. Various procedures have been developed to supply DCs with TAs. The exposure of DCs to exogenous antigens is mainly associated with MHC-class II presentation and the induction of helper T-lymphocyte dependent humoral immunity. Antigen loading by transfer of genes into DCs and intracellular transgene expression targets processed peptides to MHC-class I presentation and CTL induction.

Transfer of mp53-transduced DCs triggered systemic immunity and immunological memory against MethA tumors, most likely through the induction of specific immune effector cells. Immunization was most efficient with p53M234I transduced DCs. The mutation in codon 234 of mp53, resulting in a methionine to isoleucine substitution, is associated with the formation of a new anchor position within the MHC-class I pocket and thus might result in the presentation of a new tumor-specific antigen for CTL (48). Correspondingly, isoleucine at position 3 in K[d]-restricted non-amers is a far more frequent residue then methionine. However, p53C132F/E168G transduced DCs also induced anti-tumor immunity, although these mutated sites have not been predicted to be relevant for MHC-class I presentation. The relevant epitope, which triggers MethA tumor rejection in animals vaccinated with D2SC/1–p53C132F/E168G has still to be identified.

Cancer cells, under the onslaught of chemotherapeutic agents or immune cells, evolve and acquire resistant phenotypes. Inherent, acquired and adaptive resistance mechanisms have been identified. We found that MethA cells can escape the mp53 specific immune response by downregulation of p53 expression. Immune tolerance and resumption of tumor cell growth after the induction of a TA specific immune response have been observed before (49). It will be interesting to analyze the MethA tumors with downregulated p53 expression for alterations in transformation parameters and drug responsiveness.

The emergence of tumor escape variants can possibly be avoided by the use of vaccines, which present multiple TAs. We used fusions of tumor cells with DCs to obtain fusion hybrids. These cells possibly express all TAs and combine it with the immune stimulatory capacities of APCs (17, 42). Immunity might be enhanced because different TAs may induce different immune effector mechanisms (50, 51). Upon establishment of a vaccination protocol, the generation of tumor/DC-fusion hybrids may thus result in a simple form of personalized anti-cancer treatment.

Fusion hybrids induced a marked humoral immune response *in vivo*, a tumor-specific proliferative T-cell response *in vitro*, and prevented the emergence of tumor escape variants. Obviously, the tumor/DC-fusion hybrids are more effective therapeutic tools than single TA encoding vaccines. The broad and robust induction of specific immune effector mechanisms might be explained by the presentation of an increased number of TAs, some of them with stronger immune stimulatory capacity than mp53. Indeed, we observed the clonal expansion of multiple antigen-specific B-cells.

Immune responses induced by fusion hybrids have mainly been associated with the induction of tumor-specific CTLs (51). We additionally observed a marked humoral immune response, which could contribute to efficient anti-tumor responses. Strong humoral responses usually reflect large tumor burden and the failure of anti-tumor immune responses. However, IgG antibodies could also lead to opsonization, Fc receptor dependent internalization, processing, and antigen presentation in DCs and thus might promote tumor rejection. MethA specific polyclonal B-cell activation was only found in mice resistant to tumor growth upon fusion hybrid vaccination. However, athymic nude mice were not protected. If this is due to the lack of helper T-lymphocyte dependent B-cell stimulation remains to be investigated.

Induction of long-lasting protective immunity is the primary aim in vaccination against cancer. The cytotoxic immune response is the pivotal element for persistent elimination of tumors (19). We demonstrated a strong anti-tumor response mediated by CD3^+^ T-lymphocytes from fusion hybrid vaccinated mice. Our depletion studies confirmed that both cytotoxic and helper T-lymphocytes were essential for tumor rejection. Most known TAs are restricted to MHC-class I presentation, but MHC-class II restricted TA epitopes have also been identified. TAs might enable both CD4^+^ and CD8^+^ T-cell activation.

The therapeutic efficiency of DCs fused with autologous tumors has been associated with increased TA presentation and efficient co-stimulation of immune effector cells (17, 42). In our experiments, various DC-fusion hybrid clones were characterized by different expression profiles of antigen presenting and co-stimulatory molecules that did not necessarily reflect vaccination efficacy. Obviously, in some clones expression of MHC complexes and B7 co-stimulatory molecules was not sufficient to induce their rejection by the immune competent host. DCs are potent activators of regulatory CD4^+^ CD25^+^ Foxp3^+^ regulatory T-cells that might downregulate an efficient anti-tumor response (52, 53). Indeed, D2SC/1 cells are able to modulate regulatory T-cell activity under certain conditions (54). Additionally, we observed that MethA tumor cells are FasL^+^ and T-cells, cultured in the presence of MethA tumor cells or MethA conditioned medium, show decreased viability or reduced proliferative capacity, which might contribute to the limited vaccination potential of some fusion hybrid clones (Matjaz Humar, unpublished observation). On the other hand, lack of MHC II, B7-1, and B7-2 expression did not necessarily lead to tumor escape, indicating that other yet unidentified immune stimulatory mechanisms participate in tumor cell rejection. These additional anti-tumor response mechanisms and the probability that some hybrid clones may favor tumor escape by activating regulatory T-cells or by expressing pro-apoptotic mediators need to be elucidated in further experiments.

Vaccination against single TAs may induce an initial therapeutic benefit, but eventually could yield variant tumor cells evading the effector immune cells. Clinical trials based on the induction of immune responses against a specific TA did not necessarily prolong patient survival (55, 56). Based on our results it would be worthwhile to investigate whether tumor recurrence in those patients correlated with immune escape due to antigen downregulation.

In conclusion, multi-antigen tumor vaccines have greater therapeutic potential then DCs that present only one specific tumor-antigen. Tumors often show heterologous protein expression and selective pressure to variants with specifically reduced tumor-antigen levels will be only maintained by multi-antigen directed tumor vaccines. In addition, multi-antigen tumor vaccines provide enhanced immunity because different TAs may induce different immune effector mechanisms, resulting in a broad immune response, and synergistic cross talk of diverse immune effector mechanisms. Moreover, the generation of DC/tumor fusion hybrids avoids the complex identification and characterization of individual immune-stimulating tumor-antigens. Thus, the administration of DC/tumor fusion hybrids may provide a simple and effective form of personalized anti-cancer treatment.

## Author Contributions

Matjaz Humar generated the cellular tumor vaccines, performed the immunizations, analyzed the anti-tumor responses, and drafted the manuscript. Marc Azemar contributed to the treatment and analysis of nude mice. Martina Maurer performed the X-Gal staining and supported the tumor cell transplantation experiments. Bernd Groner supervised and coordinated the experiments and drafted and revised the manuscript. All authors have read and approved the final manuscript.

## Conflict of Interest Statement

The authors declare that the research was conducted in the absence of any commercial or financial relationships that could be construed as a potential conflict of interest.

## Supplementary Material

The Supplementary Material for this article can be found online at http://www.frontiersin.org/Journal/10.3389/fonc.2014.00270/abstract

## Abbreviations

CD, clusters of differentiation; CTL, cytolytic T-lymphocytes; DC, dendritic cells; FITC, fluorescein isothiocyanate; GM-CSF, granulocyte macrophage colony-stimulating factor; HRP, horseradish-peroxidase; IL, interleukin; i.v., intravenous; MHC, major histocompatibility complex; mp53, mutant p53; s.c., subcutaneous; TA, tumor antigen.
