# Supplementary material for: Adaptive Resistance to Immunotherapy Directed Against p53 Can be Overcome by Global Expression of Tumor-Antigens in Dendritic Cells
Source: Front Oncol. 2014 Oct 6;4:270. doi: 10.3389/fonc.2014.00270 (PMC4186483; doi:10.3389/fonc.2014.00270)
Supplement: Supplementary file 1 [file Image1.PDF]

Supplemental Figure 1

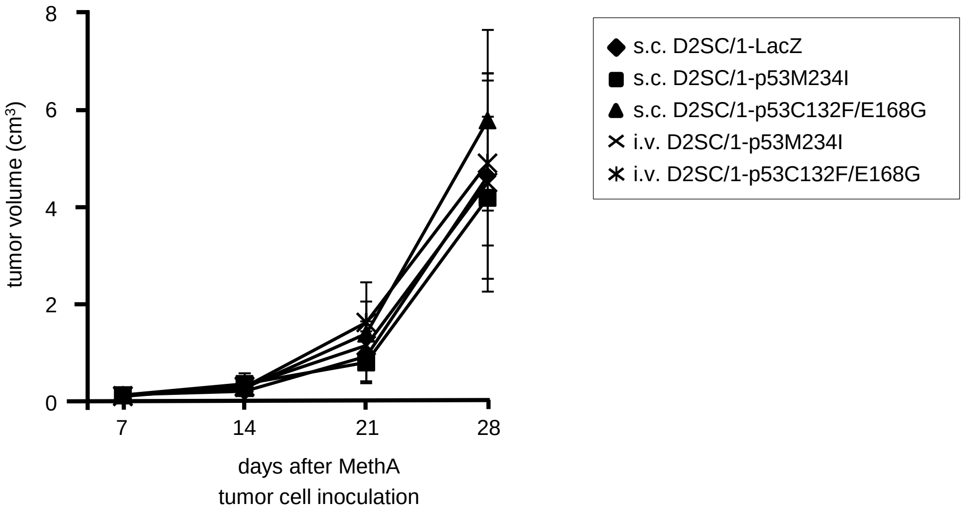

Supplemental Figure 1 MethA tumor growth on mice, immunized with D2SC/1-LacZ or D2SC/1-p53. 5 x 10^6 MethA tumor cells were transplanted into the left flank of Balb/c mice 60 days after onset of immunization with 5 x 10^6 irradiated (50 gray) D2SC/1-p53M234I (■,×) or D2SC/1-p53C132F/E168G (▲,✱) as previously described in Figure 3. The cellular vaccine was injected either subcutaneously into the right flank (s.c.) or intravenously into the tail vain (i.v.) of the animals. No statistically significant difference in tumor growth kinetics could be observed versus D2SC/1-LacZ (♦) immunized control mice. Mice, that rejected MethA tumor cells upon immunization are not shown.
